# CO₂ angiography offers clinical advantages over iodinated contrast in endovascular aneurysm repair: a systematic review and meta-analysis

**DOI:** 10.1186/s12893-025-03329-2

**Published:** 2025-11-28

**Authors:** Guohang Shen, Yupei Dai, Kaiyong Wang, Haobo Zhang, Yaxu Xin, Yang Chen, Zongxin Li, Feng Li, Fengli Gao

**Affiliations:** 1 Department of Clinical Medicine, Ningxia Medical University, Yinchuan, 750004 Ningxia Hui Autonomous Region PR China; 2 General Hospital of Ningxia Medical University, 804 Shengli South Street, Yinchuan, Ningxia Hui Autonomous Region, Yinchuan, 750004 PR China

**Keywords:** CO_2_, EVAR, ICM, Meta-Analysis, Vascular interventional therapy

## Abstract

**Objective:**

This study evaluates the safety, efficacy, and clinical outcomes of carbon dioxide (CO₂) versus iodinated contrast media (ICM) in endovascular aneurysm repair (EVAR), an area where comprehensive combined imaging and clinical assessments have yet to be explored.

**Methods:**

A comprehensive literature search was conducted in PubMed, Embase, and Scopus from their inception to May 2025, specifically targeting studies published since 2012 that compared contrast media in EVAR. Outcome measures—including operative time, fluoroscopy time, procedural success (defined as postoperative restoration of blood flow), renal function, incidence of acute kidney injury (AKI), and type II endoleak—were analyzed using a generalized linear mixed model.

**Results:**

Compared with ICM, CO₂ angiography showed no significant differences in procedural success or operative time (both *P* > 0.05), while fluoroscopy duration was slightly longer (WMD = 2.75, *P* < 0.001). CO₂ angiography was associated with markedly reduced ICM exposure (WMD = − 57.24, *P* < 0.05) and showed potential renal benefits, including higher eGFR (WMD = 0.42, *P* < 0.001) and lower serum creatinine at 1 month (WMD = − 0.09, *P* = 0.04). The incidence of postoperative acute kidney injury was also lower in the CO₂ group (1.3% vs. 2.8%; OR = 0.43, *P* = 0.05). Moreover, CO₂ angiography showed a reduced incidence of type II endoleaks (OR = 0.57, *P* = 0.04), although further confirmation in larger prospective studies is warranted.

**Conclusion:**

Based mainly on observational data with limited prospective studies, CO₂ angiography in EVAR shows comparable intra-procedural visualization and technical success to ICM. Some studies suggest it may reduce perioperative AKI risk or provide short-term renal protection. However, current evidence is insufficient to establish its equivalence across all patient populations. Therefore, its use should be individualized based on factors like renal function and anatomy or considered alongside low-dose ICM.

## Introduction

iodinated contrast media (ICM) are widely used in endovascular procedures but are associated with nephrotoxicity and allergic reactions. In contrast, carbon dioxide (CO₂) provides a non-nephrotoxic, iodine-free alternative with rapid metabolism and no allergic risks, making it an ideal choice for patients with renal dysfunction or iodine allergies—particularly in endovascular aneurysm repair (EVAR) [1–3]. While ICM remains the standard, CO₂ is increasingly recognized as a viable alternative, especially in complex cases where reduced doses of ICM (< 50 mL) are combined with CO₂ to minimize nephrotoxic risks. The utility of CO₂ has been validated in various vascular interventions, including hepatic venography, arteriovenous diagnostics, and transjugular intrahepatic portosystemic shunt (TIPS) procedures. However, its application in EVAR is limited by concerns regarding manual injection and potential complications, such as mesenteric ischemia and gas embolism. While these complications are rare, studies have reported their occurrence in a small percentage of cases, with mesenteric ischemia occurring in approximately 0.5–2.5% of patients and gas embolism in less than 1%. These risks, though infrequent, highlight the need for caution and further investigation in clinical settings [1, 2, 4].

Notably, systematic reviews and meta-analyses have explored the comparative efficacy of ICM and CO₂ in vascular procedures beyond EVAR. A meta-analysis by Ghumman et al. (2017) focusing on peripheral angiography demonstrated that CO₂ use was associated with a lower risk of contrast-induced acute kidney injury (CI-AKI) compared to ICM (relative risk = 0.47, 95% CI: 0.27–0.82), with no significant differences in procedural success rates [1, 3, 5–7]. Similarly, Wagner et al. (2022) conducted a systematic review including peripheral and visceral vascular interventions, confirming that CO₂ reduced CI-AKI incidence (odds ratio = 0.38, 95% CI: 0.21–0.69) and iodine exposure, though it noted longer fluoroscopy times in CO₂ groups due to technical learning curves [8–11]. These studies collectively highlight CO₂’s renal safety profile across vascular specialties. In terms of imaging performance, a review by Sharafuddin and Marjan (2017) summarized that CO₂ angiography achieves comparable diagnostic accuracy to ICM in low-flow vasculature (e.g., iliac and renal arteries) when combined with modern imaging techniques, despite occasional limitations in large-vessel opacification [8]. However, data specifically comparing CO₂ and ICM in EVAR—particularly regarding long-term outcomes such as endoleak—remain fragmented, with no comprehensive meta-analysis synthesizing evidence from both randomized and observational studies. Recent advances in automated CO₂ injection systems (e.g., the Angiodroid SRL system) have enhanced precision and safety, making CO₂-guided EVAR more feasible for patients with renal impairment [12, 13]. These systems utilize automated pressure control to maintain stable injection rates, minimizing gas leakage and optimizing imaging quality. CO₂-guided EVAR has shown promise in preventing contrast-induced nephropathy (CIN) and reducing ICM usage, with standardized zero-ICM protocols proving safe in select cohorts [14]. However, challenges persist in complex anatomical sites, where adjunctive techniques (e.g., patient repositioning, selective catheterization) are required to optimize imaging [15]. The primary objective of this systematic review and meta-analysis is to comprehensively compare the safety and efficacy of CO₂ versus ICM in EVAR, with a focus on renal outcomes (eGFR, serum creatinine, AKI incidence), procedural metrics (fluoroscopy time, operative success), Surgical success rate and post-procedural complications (type II endoleak), to provide evidence-based guidance for clinical practice.

## Methodology

The Preferred Reporting Items for Systematic Reviews and Meta-Analyses (PRISMA) guidelines were followed in the reporting of the results of this systematic review [16]. The results of this systematic evaluation have been formally registered in the PROSPERO database (registration number: CRD42024623666).

### Search strategy and eligibility criteria

To ensure a comprehensive and unbiased selection of relevant literature, two independent reviewers (A and B) systematically searched four major databases—PubMed, Cochrane Library, Web of Science, and Embase—up to May 2025. No language restrictions were imposed to avoid selection bias. The search strategy used Medical Subject Headings (MeSH) and free-text terms related to ‘CO₂,’ ‘ICM media (ICM),’ and ‘endovascular aneurysm repair (EVAR),’ combined with Boolean operators for an optimal search. ' For example, the PubMed search query was constructed as follows: ((((EVAR) OR (aortic aneurysm EVAR)) OR (endovascular aneurysm repair)) AND (((carbon dioxide) AND (carbon dioxide contrast agent)) AND (CO2))) AND ((((((ICM) OR (ICA)) OR (iodine contrast agent)) OR (ICM media)) OR (iodine contrast))).

To maximize sensitivity and precision, synonyms, variant spellings, and related terminology were incorporated. After deduplication, titles and abstracts were screened independently by reviewers A and B using EndNote software. Any discrepancies in study inclusion were resolved through discussion with a third reviewer (C), ensuring methodological rigor and consensus-based decision-making.

### Inclusion and exclusion criteria

Eligibility criteria were developed based on the PICOS framework (Population, Intervention, Comparison, Outcomes, Study design) to ensure methodological rigor and relevance [17, 18]. The key elements included: Population (P): Patients undergoing EVAR. Intervention (I): Angiography using CO₂ as the contrast agent. Comparison (C): Angiography using conventional ICM. Outcomes (O): Procedure time, fluoroscopy time, procedural success, postoperative renal function (eGFR, serum creatinine), incidence of AKI, and type II endoleak—capturing safety, efficacy, perioperative performance, and follow-up outcomes. Study Design (S): Randomized controlled trials (RCTs) and cohort studies. Inclusion criteria comprised studies directly comparing CO₂ and ICM in EVAR, with outcome measures covering intraoperative safety and postoperative clinical endpoints. Exclusion criteria encompassed duplicate publications; uncontrolled or non-comparative designs (e.g., case reports, reviews, conference abstracts, meta-analyses, single-arm studies); studies involving non-EVAR populations (e.g., aneurysms at other anatomical sites); and studies exclusively focused on patients with renal insufficiency or ICM hypersensitivity.

### Data extraction and quality assessment

Two independent reviewers (A and B) extracted data from studies that met the inclusion criteria. Baseline variables included: publication year, sample size, sex, age, comorbidities (e.g., diabetes, hypertension), chronic kidney disease stage, aneurysm location and diameter, indications for CO₂ use, injection method (automated vs. manual), stent type, branch vessel embolization status, and preoperative renal function. Outcome variables included: operative time, fluoroscopy time, volume of contrast agent used, procedural success rate, renal function indices at various postoperative time points, incidence of AKI, types of endoleaks, other complications (e.g., gas embolism), follow-up duration, and loss to follow-up rate. When multiple datasets were reported, the most comprehensive data were included. Median values were converted into means and standard deviations for meta-analysis [19, 20].

The risk of bias was independently assessed. For RCTs, the Cochrane Risk of Bias Tool was used, covering domains including selection, performance, detection, attrition, reporting, and other biases (the latter referring to baseline imbalances). For cohort studies, the Newcastle–Ottawa Scale (NOS) was applied, evaluating selection, comparability, and outcome assessment. Studies with NOS scores ≥ 5 (out of 9) were considered of moderate to high quality. All discrepancies in data extraction or quality assessment were resolved through discussion with a senior reviewer (D) [16–21].

### Data analysis and statistical methods

Data analysis was conducted using Review Manager version 5.4. For continuous variables, outcomes were synthesized using the weighted mean difference (WMD) with 95% confidence intervals (CIs), ensuring standardized comparisons. When scales differed across studies, the standardized mean difference (SMD) was applied. Dichotomous variables were analyzed using odds ratios (ORs) and 95% CIs. For studies reporting medians and interquartile ranges, appropriate statistical methods were used to convert these into means and standard deviations for meta-analysis inclusion [17, 22]. The Cochran-Mantel-Haenszel method was employed for dichotomous data, while continuous data were analyzed using the inverse variance method. Heterogeneity was assessed using I² values. A fixed-effect model was applied when I² < 50% and *P* >0.1; a random-effects model was used when I² ≥ 50% to account for significant heterogeneity. This approach maximized result reliability, adjusting for study variability [23].

### Sensitivity analysis

To evaluate the robustness and reliability of the findings, sensitivity analysis was performed by sequentially excluding each study and recalculating summary statistics. Consistent results across exclusions indicated high robustness. Significant deviations, however, suggested that the excluded study had a notable impact on the overall outcomes, necessitating further examination. Sensitivity analysis was not conducted when fewer than three studies were included, in accordance with established guidelines [18, 24].

### Assessment of publication bias

As statistical power may be limited when including 10 or fewer studies, publication bias could not be accurately assessed. During the assessment process, if there is disagreement among the reviewers, consensus is reached through thorough discussion to ensure the objectivity and accuracy of the assessment results [7].

## Result

### Results of the literature search

**Fig. 1:**
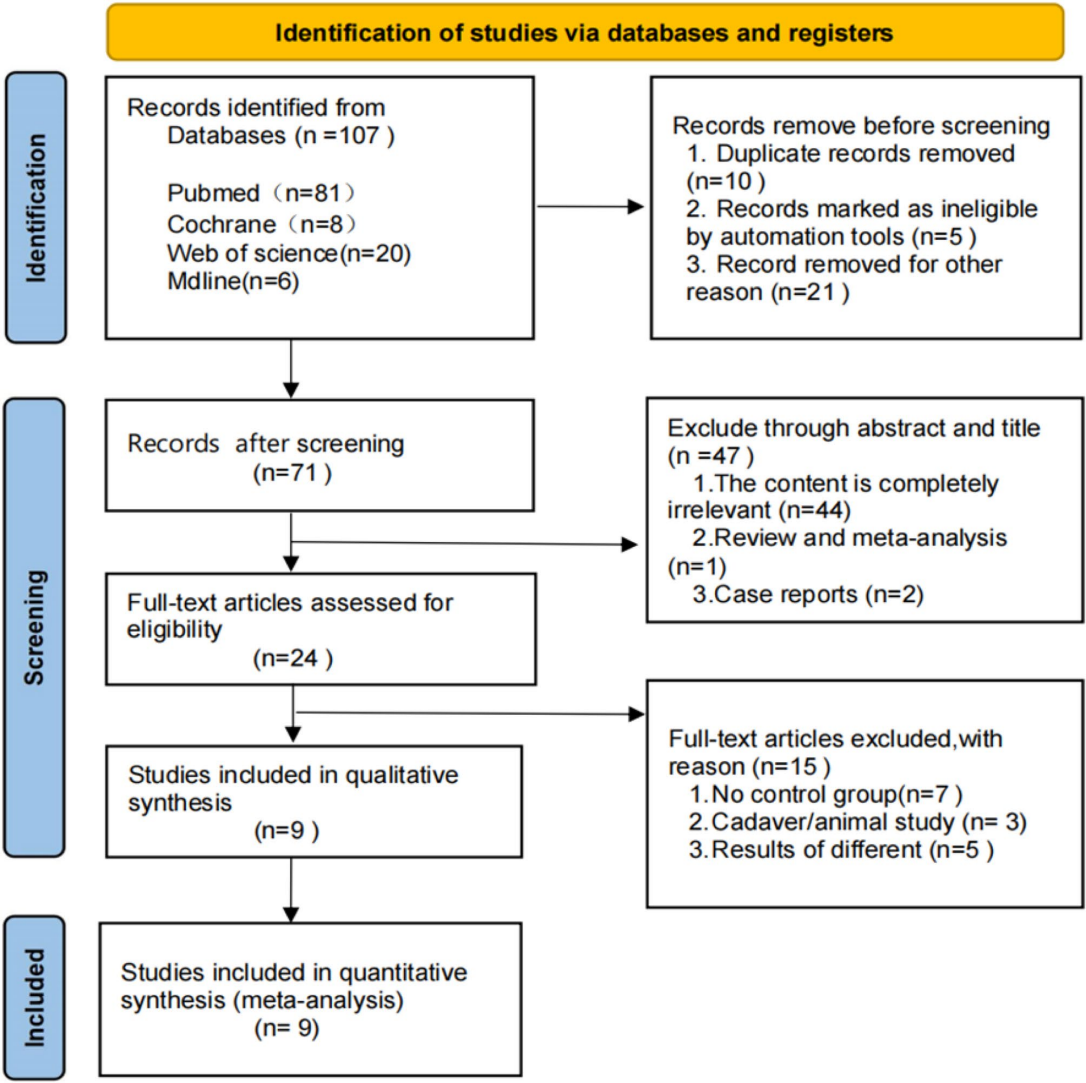
Research options flow chart

A total of 107 articles were initially retrieved from English-language databases. After removing duplicates and excluding ineligible studies, 71 articles remained. Screening by titles and abstracts reduced this number to 24. Following a full-text review, 15 articles were excluded for reasons specified in Table 1. Ultimately, nine studies met the inclusion criteria for final analysis [3, 9, 12, 14, 25–29], as shown in the PRISMA flow chart (Fig. 1).

**Table 1:** Main characteristics of the included studies

	Intervention		Mendes et al.	Takeuchi et al.		Chisci et al.	Vacirca et al.	Quaglino et al.	Vaccarino et al.	Busutti et al.	Criado et al.	Villani et al.
Publication year period (year)			2017 2012–2014	2018 2012–2016	2024 2023–2024		2022 2016–2019	2024 2020–2023	2024 2013–2019	2023 2016–2019	2012 2007–2011	2025 2019–2023
Study design			RCT	RCS	RCS		RCS	RCS	RCS	RCS	RCS	RCS
Patient numbers (n)		ICM	20	351	53		249	49	21	42	22	21
		CO_2_	16	30	240		72	52	23	72	22	21
Gender (male/female)		ICM	17/3	282/69	47/6		223/26	48/1	19/2	22/0	94/20	20/1
		CO_2_	13/3	25/5	209/31		67/5	48/4	22/1	22/0		20/1
Mean age (year)(mean ± SD)		ICM	70.6 ± 9.3	76 ± 9.0	79.29 ± 7.62		77.2 ± 7.5	77.35 ± 6.87	68.92 ± 7.15	78.5 ± 6.5	71.8 ± 8.4	77.86 ± 6.07
		CO_2_	71.5 ± 6.4	76 ± 7.3	77.05 ± 7.45		76.7 ± 8.2	76.93 ± 6.86	72.07 ± 7.11	75 ± 9.75		80,48 ± 4.81
Hypertension (n/%)		ICM CO_2_	18/90 10/62.5	279 (81) 27 (93)	53 (100) 205 (85)		217 (87.1) 60 (83.3)	37 (75.5) 46 (88.5)	12 (57) 13 (56)	NR NR	NR NR	18 (85.7) 18 (85.7)
Preoperative eGFR (mL) (mean ± SD)		ICM CO_2_	NR NR	63 ± 18 30 ± 15	62.29 ± 30.48 70.70 ± 25.35		71.5 ± 16.2 66.4 ± 14.4	NR NR	83.90 ± 39.75 75.16 ± 34.76	70 ± 17 73 ± 21	NR NR	43.0 ± 9.09 39.71 ± 9.01
Preoperative Cr(mg/dL)(mean ± SD)		ICM CO_2_	0.77 ± 0.38 0.61 ± 0.36	0.92 ± 0.33 2.02 ± 1.02	1.17 ± 0.45 1.07 ± 0.29		1.0 ± 0.6 1.2 ± 0.8	NR NR	0.90 ± 0.2 0.93 ± 0.3	NR NR	NR NR	1.60 ± 0.24 1.73 ± 0.36

*RCT* Randomized controlled study, *RCS* retrospective study, *Cr* Creatinine, *ICM* Iodine contrast medium, *CO*_2_ carbon dioxide, NR Not Reported

### Study characteristics and quality

Nine studies were included, comprising one RCT and eight retrospective cohort studies [3, 9, 12, 14, 25–29]. A total of 1,376 patients were included across nine centers, with 548 in the CO₂ group (496 received CO₂ combined with minimal ICM, and 52 received CO₂ alone) and 828 in the ICM group. No significant differences were found between the groups in terms of sex, age, and gender distribution (*P* >0.05), indicating comparable baseline characteristics. Table 2 outlines the basic characteristics of the included studies, while Table 3 summarizes intraoperative and postoperative evaluation indices.

**Table 2:** Results from nine studies

		Mendes et al.	Takeuchi et al.	Chisci et al.	Vacirca et al.	Quaglino et al.		Vaccarino et al.	Busutti et al.	Criado et al.	Villani et al.
Operative time (minutes)(mean ± SD)	ICM	172 ± 39	171 ± 86	120 ± 60	60.29 ± 9.96	54 ± 13	179 ± 60		NR	193 ± 9	NR
	CO_2_	179 ± 26	172 ± 68	88 ± 44	89.72 ± 15.04	75.±35	181 ± 87		NR	165 ± 6	NR
Fluoroscopy time (minutes) (mean ± SD)	ICM	30 ± 11	49 ± 37	14 ± 7	NR	12 ± 4	50 ± 33		NR	NR	19.29 ± 14.37
	CO_2_	30 ± 9	43 ± 29	20 ± 13	NR	14 ± 5	50 ± 30		NR	NR	23.45 ± 10.95
Success rate of surgery (n)(%)	ICM	NR	350(99.7)	NR	NR	49(100)	21(100)		20(91)	42(100)	21(100)
	CO_2_	NR	30(100)	NR	NR	52(100)	22(95.6)		21(95)	72(100)	21(100)
Postoperative acute kidney injury rate (n)(%)	ICM	NR	10(2.8)	5(9)	2(0.8)	0	1 (4.8)		NR	NR	1 (4.8)
	CO_2_	NR	1(3)	2(0.8)	0	0	2 (8.7)		NR	NR	0
Postoperative type II Endoleak rate (n)(%)	ICM	1(5)	73(22)	7(13)	NR	7(14.3)	10.80 ± 1.60		NR	0	6(28.6)
	CO_2_	2(12.5)	4 (15)	23 (10)	NR	13(25)	15.20 ± 2.60		NR	16(22)	3(14.3)
Creatinine one month postoperative(mg/dL)(mean ± SD)	ICM	0.85 ± 0.41	NR	1.3 ± 0.45	1.15 ± 0.6	NR	NR		NR	NR	NR
	CO_2_	0.74 ± 0.38	NR	1.07 ± 0.44	1.2 ± 0.9	NR	NR		NR	NR	NR
Creatinine three months postoperative(mg/dL)(mean ± SD)	ICM	NR	0.93 ± 0.4	NR	1.22 ± 0.3	1.0 ± 0.25	1.3 ± 0.3		NR	NR	NR
	CO_2_	NR	1.79 ± 1.2	NR	1.13 ± 0.5	1.09 ± 0.37	1.0 ± 0.3		NR	NR	NR
eGFR one month postoperative (mL) (mean ± SD) ICM dose (ml)	ICM CO_2_ ICM CO_2_	NR NR 40.53 ± 22.33 7.31 ± 11.37	NR NR 55 ± 24 18 ± 15	57.35 ± 31.24 70.70 ± 25.35 NR NR	67.2 ± 6.7 69.2 ± 78 88.1 ± 9.2 52.8 ± 6.1	NR NR 100 ± 53.46 0	NR NR 105 ± 76.32 58.22 ± 29.23		63 ± 16 77 ± 20 180.2 ± 41.4 49.5 ± 34.8	NR NR 106 ± 7 37 ± 4	NR NR 65.87 ± 29.54 7.67 ± 18.88

*eGFR* estimated glomerular filtration rate, *ICM* Iodine contrast medium, *CO*_2_ carbon dioxide, *NR* not reported

**Table 3:** Risk of bias assessment

	Selection	Comparability	Outcome	Total
Takeuchi 2018	✩✩✩✩	✩	✩✩✩	✩✩✩✩✩✩✩✩
Chisci 2024	✩✩✩✩	✩	✩✩✩	✩✩✩✩✩✩✩✩
Vacirca 2022 Villani 2025	✩✩✩✩ ✩✩✩✩	✩ ✩	✩✩✩ ✩✩✩	✩✩✩✩✩✩✩✩ ✩✩✩✩✩✩✩✩
Quaglino 2024	✩✩✩✩	✩	✩✩	✩✩✩✩✩✩✩
Vaccarino 2024 Busutti 2023 Criado 2012	✩✩✩✩ ✩✩✩✩ ✩✩✩✩	✩ ✩ ✩	✩✩✩ ✩✩ ✩✩	✩✩✩✩✩✩✩✩ ✩✩✩✩✩✩✩✩ ✩✩✩✩✩✩✩

### Risk of bias assessment

**Fig. 2:**
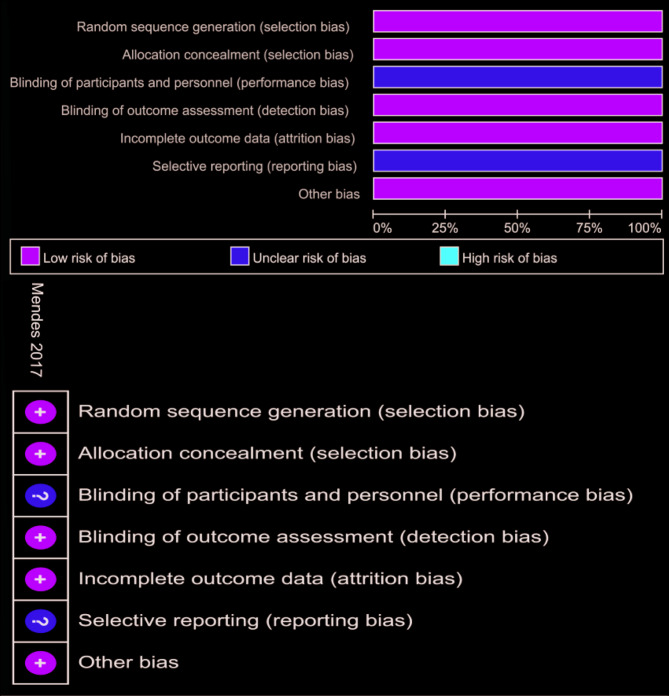
Quality of RCTs according to the Cochrane Collaboration Manual. Red: High risk; Yellow: Unclear risk; Green: Low risk

The quality and risk of bias of the nine included studies were assessed based on the evaluation criteria described in the Methods and Materials section. One RCT was evaluated according to the Cochrane Handbook, and the results indicated that this study was of good quality (Fig. 2). The remaining eight retrospective cohort studies were assessed for quality using the Newcastle-Ottawa Scale (NOS) and for risk of bias using the ROBINS-I tool. The evaluation results showed that all eight studies had NOS scores exceeding 7 (Table 4), indicating that all included studies met the criteria for high-quality research. This provides a reliable data foundation for subsequent analyses [17, 24, 29, 30].

**Table 4:** Evaluation of the risk of bias in the studies included in the analysis through the use of the ROBINS-I tool

	Bias due to confounding	Bias in selection of participants	Bias in classification of interventions	Bias due to deviations from intended interventions	Bias due to missing data	Bias in measurement of outcomes	Bias in selection of the reported result		Overall risk of bias	
Takeuchi 2018	Moderate	Moderate	Low	Low	Low	Low	Moderate	Moderate		
Chisci 2024	Moderate	Low	Low	Low	Low	Low	Moderate	Moderate		
Vacirca 2022 Villani 2025	Moderate Moderate	Low Low	Low Low	Low Low	Low Low	Low Low	Low Low	Moderate Low		
Quaglino 2024	Moderate	Low	Low	Low	Low	Moderate	Low	Moderate		
Vaccarino 2024 Marco 2023 Criado 2012	Moderate Moderate Moderate	Low Moderate Moderate	Low Low Low	Moderate Low Low	Low Low Low	Low Low Low	Low Low Low	Moderate Moderate Moderate		

### Postoperative renal function

**Fig. 3:**
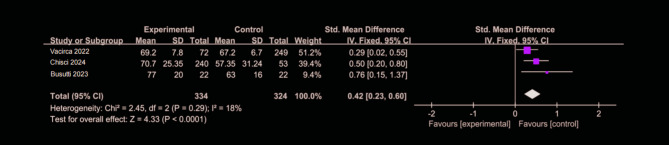
Forest plot of one-month postoperative eGFR

**Fig. 4:**
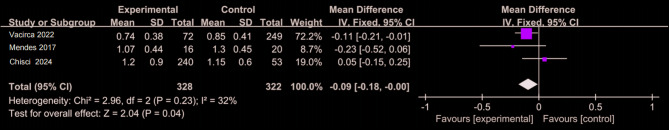
Forest plot of one-month postoperative Creatinine

**Fig. 5:**
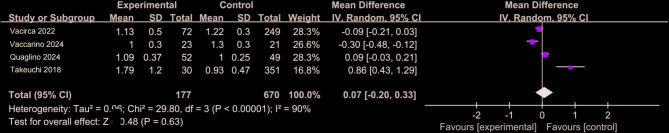
Forest plot of three-month postoperative Creatinine

Figures 3 and 4 present data on eGFR and serum creatinine levels one month postoperatively, respectively, from studies assessing postoperative renal function [3, 12, 14, 29]. The heterogeneity test revealed low to moderate heterogeneity (I² = 18% to 32%), and a fixed-effects model was applied for both outcomes. In these studies, the CO₂ group demonstrated superior renal recovery compared to the ICM group, with a significant improvement in eGFR and lower serum creatinine levels. Specifically, the WMD for eGFR was 0.42 (95% CI: 0.23–0.6, *P* < 0.001), and for creatinine, (WMD = −0.09,95% CI: −0.18 to 0.00, *P* = 0.04), indicating that CO₂ provides a significant benefit in protecting renal function in the early postoperative period. In contrast, Fig. 5 presents data on creatinine levels measured three months postoperatively. The CO₂ group comprised 177 patients, while ICM group included 670 patients [12, 26–28]. Given the high heterogeneity (I² >50%), a random-effects model was employed for analysis. There was no significant difference in clinical outcomes between CO₂ and ICM, with a (WMD = 0.07,95% CI: −0.20 to 0.33, *P* >0.05).

### Postoperative acute kidney injury and baseline creatinine levels

**Fig. 6:**
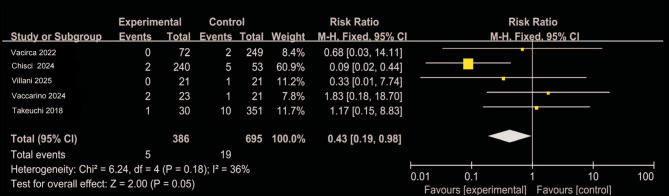
Forest plot of postoperative acute kidney injury

**Fig. 7:**
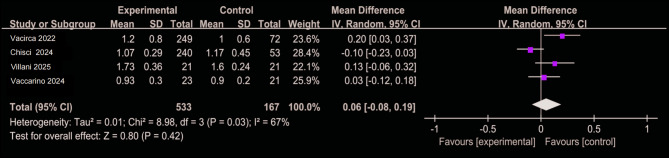
Forest plot of baseline Creatinine

Figures 6 and 7 summarize data from five studies [12, 14, 25, 26, 28], encompassing a total of 1,081 patients. The incidence of postoperative AKI was 1.3% in the CO₂ group (5/386) compared to 2.8% in the ICM group (19/695), yielding a pooled risk ratio of 0.43 (95% CI: 0.19–0.98; *P* = 0.05), suggesting a potential association between CO₂ use and reduced AKI risk. Although baseline serum creatinine levels did not significantly differ between groups when pooled, substantial heterogeneity was observed across studies (I² = 67%). Sensitivity analysis revealed that exclusion of the Emiliano (2024) study reduced heterogeneity to 10%, and further examination indicated that patients in the CO₂ group had significantly higher baseline creatinine levels, implying worse preoperative renal function. Notably, despite this disadvantage, the CO₂ group demonstrated a lower postoperative AKI incidence, which reinforces—rather than undermines—the hypothesis of a renal-protective effect associated with CO₂ angiography.

### Fluoroscopy time

**Fig. 8:**
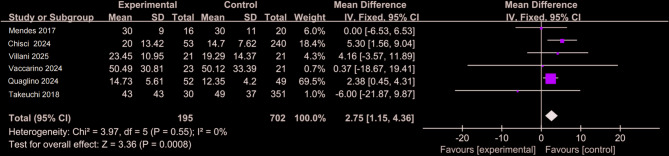
Forest plot of fluoroscopy time

During angiographic procedures, fluoroscopy time serves as a key indicator of radiation exposure to both patients and clinical staff. Figure 8 presents pooled data from five studies [14, 25–29]. involving 195 patients in the CO_2_ group and 702 in the ICM group. All studies applied ALARA-compliant dose-reduction strategies, including pulsed fluoroscopy, last image hold (LIH), collimation, and protocol optimization. With no observed heterogeneity (I² = 0%), a fixed-effects model was used. The analysis revealed a significantly longer fluoroscopy time in the CO_2_ group, with a mean difference of 2.75 units (95% CI: 1.15–4.36; Z = 3.36; *P* < 0.001). This increase likely reflects the learning curve and technical adjustments required for CO₂ angiography, which demands more precise image acquisition to ensure diagnostic adequacy [12, 25].

### Contrast medium

**Fig. 9:**
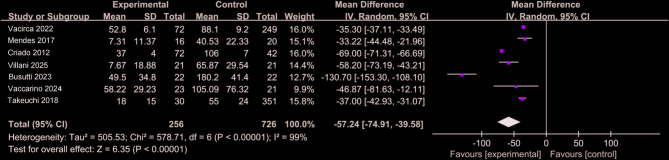
Forest plot of CO_2_ overlaid with low and sufficient concentrations of ICM

Figure 9. The CO_2_ group consisted of 256 patients, and the ICM group included 726 patients. A random-effects model revealed a significant overall effect (WMD = − 57.24; *P* < 0.05 [3, 9, 12, 25, 26, 28, 29]. The CO_2_ group, which used CO₂ with minimal ICM, showed fewer ICM-related complications than the full-dose ICM group. However, substantial heterogeneity was observed across studies, potentially due to variations in contrast agent types, dosing protocols, and imaging techniques. This limits the generalizability of the pooled result and suggests that findings should be interpreted with caution.

### Surgical time and surgical success rate

**Fig. 10:**
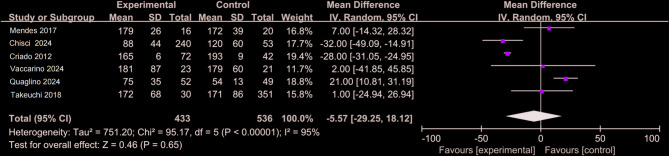
Forest plot of surgical time

**Fig. 11:**
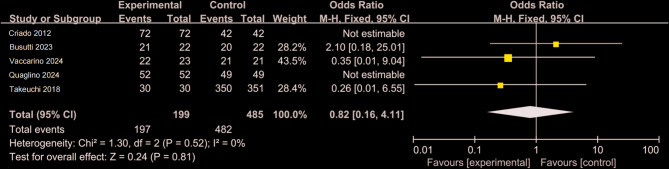
Forest plot of surgical success rate

Figure 10 presents a comparative analysis of 969 patients, including 433 in the CO₂ group and 536 in the ICM group [9, 14, 26–29]. Significant heterogeneity was observed in surgical time (I² >50%), and therefore, a random-effects model was applied (WMD = −0.17, 95% CI: −29.25 to 18.12, *P* >0.05). No statistically significant difference was found between the two groups. Figure 11 summarizes surgical success rates from five retrospective studies involving 684 patients (199 in the CO₂ group and 485 in the ICM group) [3, 9, 26–28]. Heterogeneity testing indicated no significant variation (I² = 0), supporting the use of a fixed-effects model. The studies by Criado (2012) and Quaglino (2024) reported 100% success rates in both groups, resulting in zero variance, which precluded valid weight calculation for these datasets. After pooling the available data, no statistically significant difference was identified in the success rate of EVAR between the CO₂ and ICM groups (WMD = 0.82, 95% CI: 0.01–6.55, *P* >0.05).

### Type II endoleak post-surgery

**Fig. 12:**
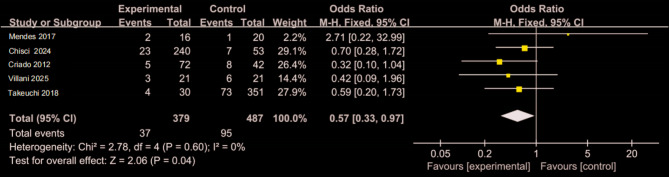
Forest plot of postoperative type II Endoleak

Figure 12 presents data from five studies, with 379 patients in the CO₂ group and 487 in the ICM group [9, 14, 25, 28, 29]. At the 1-month postoperative timepoint, the incidence of type II endoleak was slightly lower in the CO₂ group (9.76%) than in the ICM group (19.5%) (OR 0.57, 95% CI: 0.33–0.97, *P* = 0.04).

## Discussion

This meta-analysis incorporated nine studies with a total of 1,376 patients, which, to our knowledge, is the first such study to compile data from multiple sources in the field of EVAR. Quality assessment indicated that most of the included studies demonstrated reasonable methodological rigor, though some limitations remain. This analysis, which includes both RCT and retrospective studies (RCS) [3, 9, 12, 14, 25, 27–29], suggests the advantages of CO₂ as a contrast medium in EVAR and provides supportive evidence for its clinical safety and efficacy. Specifically, CO₂ was associated with a reduced incidence of AKI and renal impairment, likely due to its non–nephrotoxic profile, lower osmolality, and absence of iodine–induced oxidative stress [31]. Moreover, the use of CO₂ angiography may be associated with a lower incidence of postoperative type II endoleaks, suggesting a potential improvement in vascular outcomes. Not only may it benefit patients with renal insufficiency or iodine allergy, but it could also serve as a potential alternative in the broader population, pending further validation. The combined use of CO₂ and low-dose ICM enhances image quality in low-flow regions while minimizing nephrotoxicity and allergic reactions, offering a safer strategy for complex cases [7, 32]. This approach is particularly beneficial for patients with CKD stage 3–4, in whom even mild AKI episodes may hasten progression to end-stage renal disease (ESRD) [33].

The operation time and surgical success rates in the CO₂ group were comparable to those in the ICM group, indicating no significant differences in procedural outcomes. The incidence of AKI, a key focus of this study, was lower in the CO₂ group (1.3%) compared to the ICM group (2.8%). The few AKI cases in the CO₂ group were likely due to pre-existing renal dysfunction or perioperative hemodynamic instability rather than the contrast agent itself [7, 11]. Notably, none of the patients in the CO₂ group required postoperative hemodialysis, further emphasizing the renal safety of CO₂ as a contrast agent, especially for patients with renal dysfunction. One month postoperatively, patients receiving CO₂ showed improved eGFR, underscoring CO₂’s potential to promote faster recovery and better preservation of renal function, contributing to favorable patient outcomes [12, 26–28]. Fluoroscopy time, indicative of radiation exposure, was reduced as operator experience with CO₂ imaging techniques increased. The learning curve for CO₂ angiography has led to optimized catheter positioning, CO₂ injection timing, and image acquisition parameters, thus improving procedural efficiency and minimizing unnecessary radiation exposure [8, 9, 12, 13, 34, 35].

The combination of CO₂ and a minimal dose of ICM enhances image quality, addressing CO₂’s limitations, such as suboptimal opacification in low-flow or gravity-dependent vessels and limited visualization of complex vascular anatomies. This hybrid approach significantly reduces ICM volume, providing a safer and more effective imaging strategy, particularly for patients at increased risk of contrast-related complications [8, 29, 34, 36]. For challenging anatomical structures, such as suprarenal aortic aneurysms, using CO₂ with low-dose ICM (< 50 mL) strikes an optimal balance between safety and image quality. This study contributes to the emerging evidence on the synergistic effects of CO₂ and low-dose ICM, suggesting potential benefits in addressing imaging challenges in complex anatomies, such as interference from transverse colon gas or high BMI, while helping to limit iodine exposure. This hybrid imaging strategy appears to reduce the use of contrast agents while maintaining acceptable image quality in most reported cases [26]. Recent prospective studies, including those by Rogers et al., further support this approach, showing an 82% success rate for zero-iodine EVAR, emphasizing the clinical viability of combining CO₂ with low-dose ICM [14]. This consistency underscores the potential of this approach to revolutionize contrast-enhanced imaging protocols, particularly for complex and high-risk patient populations [14, 15, 34, 35].

The lower incidence of type II endoleaks observed in the CO₂ group may be related to the distinct physicochemical and hemodynamic characteristics of carbon dioxide. Its very low viscosity (approximately 1/400th that of ICM) and high solubility (about 20 times that of oxygen) facilitate rapid displacement and diffusion, promoting uniform filling even in low-flow regions while allowing quick pulmonary elimination. These properties may minimize intraluminal pressure fluctuations and vessel wall stress during angiography, thereby contributing to more stable hemodynamics following EVAR [8, 29, 37]. Unlike ICM, CO₂ is chemically inert and does not provoke vasodilation or endothelial irritation, which can increase vascular permeability and inflammatory responses. Experimental studies have shown that CO₂ causes less endothelial disruption and fewer inflammatory reactions than iodine-based agents, supporting its potential vascular protective effect [38, 39]. In addition, the enhanced visualization of collateral vessels provided by CO₂ angiography may enable more accurate intraoperative assessment and embolization, thereby reducing the likelihood of residual retrograde filling that leads to type II endoleaks [12, 40]. Collectively, these findings provide a biologically plausible rationale for the observed reduction in type II endoleak incidence with CO₂ angiography. Nevertheless, this mechanism remains hypothetical and requires validation through dedicated mechanistic and histopathological studies [2, 3, 36–42]. The studies included in this meta-analysis span diverse patient cohorts from North America, Europe, and Asia, collectively demonstrating the efficacy of CO₂ across different populations. This provides evidence supporting the consideration of CO₂ as a contrast agent in renal protection during EVAR, particularly for high-risk patients. For cases involving complex anatomy(e.g., tortuous vessels, severe stenosis, bifurcations, heavy calcification, or distal small vessels), a standardized protocol combining CO₂ with a low-dose iodine-based contrast agent should be recommended to optimize safety and imaging quality [12]. Future research should focus on high-quality prospective randomized controlled trials to validate CO₂’s long-term efficacy and applicability in complex cases across different patient populations (Table 5). Additionally, updates to clinical guidelines and enhanced interdisciplinary collaboration will be essential to facilitate the broader adoption of CO₂ contrast agents in vascular interventions, helping to achieve precision medicine goals, improve patient safety, reduce CIN, and enhance long-term outcomes for patients undergoing endovascular treatments.

**Table 5:** Advantages and disadvantages of contrast medium

Analysis Dimension	CO₂		ICM	
	Advantages	Disadvantages	Advantages	Disadvantages
Kidney Function	Does not rely on renal excretion, safe for patients with end-stage renal disease or dialysis. Prevents nephrotoxicity and cumulative damage caused by iodine contrast agents.	No long-term safety data available (maximum follow-up of 1 year). In complex anatomy, a small amount of ICM (less than 50 mL) is required as an adjunct.	No research found indicating kidney function advantages.	ICM’s high osmolarity (especially ionic contrast medium) can lead to dehydration and necrosis of renal tubular epithelial cells, causing oxidative stress and inflammation. This damages endothelial cells and may lead to contrast-induced acute kidney injury (CIAKI).
Imaging Quality	Physical characteristics are applicable to imaging of multiple vascular beds. Combining a small amount of ICM (< 50 mL) can optimize the imaging of complex structures.	The imaging quality of complex anatomical structures is inferior to that of ICM. Approximately 18% of cases require the superposition of ICM for assistance.	Suitable for clear imaging of complex anatomical structures (e.g., branched aneurysms, adrenal tumors). No additional technical assistance required.	The high density of ICM in X-rays can produce artifacts when used in conjunction with metal stents, potentially interfering with the assessment of stent wall adherence and leakage.
Post-Surgical Safety	Safe for patients with end-stage renal disease or on dialysis. Surgical success rates are similar to those with ICM.	The initial perspective time is slightly longer than that of the ICM group (WMD = 2.75 min), a dedicated injection system is required, and there is an operational learning curve.	It can reduce the risks such as stent placement errors and vascular injuries caused by imaging blurring.	May cause anaphylactic shock, laryngeal edema, and even death during surgery.
Operability	Standardized procedures (e.g., adjusting injection sites) can overcome anatomical limitations. Automatic injection systems are continuously improving.	Technical proficiency affects the time of perspective	The operation process is mature and no special equipment is required.	In cases that require multiple angiographies (such as staged EVAR or postoperative follow - up), repeated use of ICM may lead to thyroid dysfunction due to excessive iodine load, or the dosage may be restricted due to nephrotoxicity, indirectly affecting the imaging quality.
Cost and Accessibility	Low in price, convenient to obtain, and applicable to a large range of patients	The independent application of complex cases may be restricted.	There is sufficient long-term clinical data, making it suitable for routine cases.	The price is relatively high, and the cost of pre-treatment for iodine allergy needs to be considered.
Conclusion	The non-nephrotoxic mechanism of CO₂, combined with its rapid metabolic properties (half-life of approximately 20 s), is related to its exhalation through the lungs after diffusion into the bloodstream. This prevents renal tubular damage caused by osmotic load and oxidative stress, offering a safer EVAR imaging option, particularly for patients with renal insufficiency or those requiring repeated imaging. CO₂ is increasingly likely to become more widely used in routine clinical settings as technology improves.			

### Limitations

The buoyant nature of CO₂ may lead to incomplete opacification in gravity-dependent vessels, such as the iliac bifurcation, femoral arteries, renal arteries, and coronary artery branches, particularly in obese patients or those with altered anatomy. Visualization of large or low-pressure vessels, such as suprarenal segments, can also be suboptimal. There is a low but notable risk of gas embolism, especially in patients with right-to-left shunts like patent foramen ovale, underscoring the need for careful patient selection and operator expertise.

Methodologically, this meta-analysis included only one randomized trial and eight retrospective studies, introducing potential bias and heterogeneity. The relatively short follow-up duration may have underestimated adverse events and long-term applicability. In addition, endoleaks at 1 month were mainly assessed by CT angiography, and variations in imaging protocols may have introduced inconsistency and increased exposure to ICM.

Moreover, because of limited and non-comparable data, the effect of CO₂ on other endoleak types (e.g., type I or III) could not be assessed. Potential selection bias may also exist, as CO₂ might have been preferentially used in patients with a lower anatomical risk of type II endoleak. Finally, anatomical factors—such as accessory renal arteries, lumbar arteries, and the inferior mesenteric artery—are well-recognized contributors to type II endoleak. However, most included studies lacked subgroup analyses stratified by these variations. To avoid overinterpretation, this limitation has been explicitly acknowledged in the manuscript and supported by relevant references.

## Conclusion

### Clinical implication

Current evidence indicates that CO₂ angiography could be a feasible alternative to ICM in selected EVAR patients, particularly those with impaired renal function. This non-nephrotoxic modality shows a possible association with a lower risk of CIN while maintaining acceptable diagnostic accuracy, though further high-quality studies are warranted to confirm these findings. The results of this meta-analysis indicate that CO₂ angiography may be associated with a lower incidence of postoperative type II endoleaks. Given the observed reduction in type II endoleaks, further research is essential to assess CO₂’s effects on other endoleak subtypes (e.g., type I and III) and fully evaluate its role in EVAR. Despite the limited large-scale evidence, early findings suggest that CO₂ provides comparable endoleak detection, particularly for type II endoleaks, and yields outcomes similar to ICM. CO₂’s favorable physicochemical properties—low viscosity, high solubility, and buoyancy—may underlie its hypothesized protective effects, which warrant further investigation [43, 44].

### Future directions

The effectiveness of CO₂ imaging may vary with aneurysm anatomy. Infrarenal aneurysms are generally well-visualized, while juxtarenal or suprarenal types and large sacs with thrombus or complex morphology may impair gas distribution and image clarity. These anatomical factors should guide contrast selection. Despite promising results, further research is needed to confirm the long-term renal and clinical benefits of CO₂ in EVAR. Future multicenter RCTs with extended follow-up should assess its impact on survival, complications, and quality of life. Additionally, optimizing CO₂ delivery protocols and its combination with minimal ICM use warrants investigation to support broader clinical adoption [32, 41, 43, 44].

### Implementation strategy

To further advance the clinical application of CO₂ angiography in EVAR, we recommend adopting automated injection systems, such as the CO₂MMANDER or AngioDynamics injectors, which ensure stable and regulated injection pressures (300–400 mmHg) and precisely controlled injection volumes (30–60 mL) [45], potentially enhancing both image quality and procedural safety. Compared to manual injection, automated delivery systems reduce the risk of gas embolism and improve the stability and reproducibility of vascular imaging.

Therefore, automated CO₂ injection may be considered as a preferred modality in protocol development for CO₂-EVAR and could be incorporated into the structured training curricula for vascular surgeons and interventional radiologists. Additionally, institutions are encouraged to integrate CO₂ angiography into hybrid operative strategies, in conjunction with minimal doses of iodinated contrast media (ICM < 50 mL), to further optimize patient safety, renal preservation, and procedural efficiency.

## Data Availability

The datasets used and/or analysed during the current study are available from the corresponding author on reasonable request.
